# Thermal-Induced Oxygen Vacancy Enhancing the Thermo-Chromic Performance of W-VO_2−x_@AA/PVP Nanoparticle Composite-Based Smart Windows

**DOI:** 10.3390/nano15141084

**Published:** 2025-07-12

**Authors:** Jiran Liang, Tong Wu, Chengye Zhang, Yunfei Bai, Dequan Zhang, Dangyuan Lei

**Affiliations:** 1 School of Microelectronics, Tianjin University, Tianjin 300072, China; 2 Tianjin Key Laboratory of Imaging and Sensing Microelectronic Technology, Tianjin University, Tianjin 300072, China; 3 Tianjin SYP Engineering Glass Co., Ltd., Tianjin 300409, China; 4 Department of Materials Science and Engineering, City University of Hong Kong, Kowloon, Hong Kong 999077, China; 5 Center for Functional Photonics, City University of Hong Kong, Kowloon, Hong Kong 999077, China; 6 Hong Kong Institute of Clean Energy, City University of Hong Kong, Kowloon, Hong Kong 999077, China; 7 Hong Kong Branch of National Precious Metals Material Engineering Research Centre, City University of Hong Kong, Kowloon, Hong Kong 999077, China

**Keywords:** oxygen vacancy, W-VO_2_, AA, smart windows

## Abstract

Tungsten-doped vanadium dioxide (W-VO_2_) shows semiconductor-to-metal phase transition properties at room temperature, which is an ideal thermo-chromic smart window material. However, low visual transmittance and solar modulation limit its application in building energy saving. In this paper, a W-VO_2−x_@AA core-shell nanoparticle is proposed to improve the thermo-chromic performance of W-VO_2_. Oxygen vacancies were used to promote the connection of W-VO_2−x_ nanoparticles with L-ascorbic acid (AA) molecules. Oxygen vacancies were tuned in W-VO_2_ nanoparticles by thermal annealing temperatures in vacuum, and W-VO_2−x_@AA nanoparticles were synthesized by the hydrothermal method. A smart window was formed by dispersing W-VO_2−x_@AA core-shell nanoparticles into PVP evenly and spin-coating them on the surface of glass. The visual transmittance of this smart window reaches up to 67%, and the solar modulation reaches up to 12.1%. This enhanced thermo-chromic performance is related to the electron density enhanced by the AA surface molecular coordination effect through W dopant and oxygen vacancies. This work provides a new strategy to enhance the thermo-chromic performance of W-VO_2_ and its application in the building energy-saving field.

## 1. Introduction

Tungsten-doped vanadium dioxide (W-VO_2_) has semiconductor-to-metal phase transition properties at room temperature [1,2]. It strongly shows transmittance modulation in the near infrared band, while visual transmittance keeps a constant accompanied phase transition, which has potential application in smart windows for energy saving in building [3,4,5]. The visual transmittance and solar modulation (dependent on near infrared transmittance difference) are two important performance parameters of W-VO_2_-based smart windows [6,7].

Tungsten doping decreases the phase transition temperature of VO_2_ but also worsens the performance [8]. W-VO_2_ nanoparticles dispersed into PVP show higher transmittance in a lower-temperature semiconductor state for decreasing absorption in the visual band. When a W-VO_2_ nanoparticle is in a metallic state, it has a localized surface plasmon resonance (LSPR) absorption enhancement property [9], which can increase the transmittance difference in the near IR band to improve the solar modulation [10,11]. Core-shell or multi-layer nanostructures were designed to improve the performance of smart windows, such as SnO_2_/W-VO_2_/SnO_2_ [12] and W-VO_2_@SiO_2_ [13]. These structures can increase the visual transmittance by decreasing the difference in the refractive index between W-VO_2_ and air and the solar modulation by enhancing the LSPR through separating the nanoparticles. Liang et al. proposed and fabricated a W-VO_2_@L-ascorbic acid (AA) core-shell nanoparticle structure [14]. In this structure, the AA not only decreases the difference in the refractive index but also injects electrons into W-VO_2_, which enhances the visual transmittance and solar modulation simultaneously. The thickness of AA on W-VO_2_ is important for the performance enhancement. However, it is a challenge to increase the thickness [15].

In this study, a thermal-induced oxygen vacancy was proposed to enhance the connection of W-VO_2_ nanoparticles with AA molecules and form W-VO_2−x_@AA nanoparticles to improve the thermo-chromic performance of W-VO_2_. The oxygen vacancy can be used to increase the electron density and surface molecular coordination effect between AA and W-VO_2_. The oxygen vacancy was induced in W-VO_2_ nanoparticles by thermal annealing in a vacuum. The connection of AA on the surface of W-VO_2_ nanoparticles by the hydrothermal method and the shell thickness of AA was modulated by the oxygen vacancy concentration. The solar modulation of the W-VO_2−x_@AA/PVP composite film-based smart windows reaches up to12.1%, and the visual transmittance reaches up to 67%. The thermo-chromic performance is higher than that of W-VO_2_@AA/PVP composite film-based smart windows. This work will enhance the application of W-VO_2_ nanoparticles in the building energy-saving field.

## 2. Experiments

### 2.1. Materials

W-VO_2_ powder (average diameter = 60 nm, 2 wt.%) was obtained from Hangzhou Jikang New Material Co., Ltd. (Hangzhou, China). Glacial acetic acid (>99.5%(T)) was produced by Tianjin Bohai Chemical Reagent Co., Ltd. (Tianjin, China). AA was bought from Shanghai Macklin Biochemical Technology Co., Ltd., and the purity is higher than 99.0%(T).

### 2.2. Preparation of W-VO_2−x_

Oxygen vacancy in W-VO_2_ nanoparticles was induced by thermal annealing in a vacuum. The W-VO_2_ nanoparticles were placed in a cube with an Ar environment and annealed at temperatures of 300, 350, 400, 450, and 500 °C for 30 min. The prepared samples are named T300, T350, T400, T450, and T500, and the untreated nanoparticles were named T0.

### 2.3. Preparation of W-VO_2−x_@AA

First, glacial acetic acid was dropped into deionized water to form a solution at room temperature. The volume was 10 mL and 25 mL, respectively. The solution was stirred to dissolve and ionize the glacial acetic acid completely. The stirring time was 30 min. Then, AA was added to the solution; the weight was 4.403 g. In order to dissolve the AA fully, the stirring time was 15 min, and the temperature was room temperature. Then, the annealed W-VO_2−x_ nanoparticles were added to the mixture solution. The weight was 300 mg. In order to disperse the W-VO_2−x_ nanoparticles uniformly into the mixture solution, it was sonicated for 1 h at room temperature. In order to promote the connection of AA and W-VO_2−x_ nanoparticles, the mixture was heated to 90 °C and maintained for 8 h. At last, the product was collected. The black product was centrifuged and washed with deionized water and ethanol. The samples were named TA0, TA300, TA350, TA400, TA450, and TA500, according to the annealing temperature.

### 2.4. Preparation of W-VO_2−x_@AA/PVP Composite Thin Films

The W-VO_2−x_@AA nanoparticles were dispersed into PVP and were spin-coated on the surface of glass to form W-VO_2−x_@AA/PVP composite thin films using the sol-gel method. First, 2.5 cm × 2.5 cm transparent glass substrates were cleaned sequentially with acetone, anhydrous ethanol, and deionized water. Next, PVP was dissolved in anhydrous ethanol in a vial to form a PVP solution. The weight of PVP is 600 mg, and the volume of anhydrous ethanol is 3 mL. To prevent ethanol evaporation, the vial was sealed with plastic wrap. The solution was sonicated for 30 min to make sure the PVP dissolved completely. Then, 20 mg of the W-VO_2−x_@AA powder was added to the PVP solution. The mixture was then sonicated for 60 min at 40 °C. After sonication, the mixture solution was stirred for 4 h, placing it for 24 h; then, the suspension was obtained. The suspension was spin-coated on the surface of glass substrate. The volume of suspension was 140 µL. The spin speed was 800 rpm, maintained for 10 s, and then increased to 2000 rpm for 20 s. After spin-coating, the composite film was placed in a constant temperature oven and dried at 60 °C for 10 min.

### 2.5. Characterization

The crystal lattice structure of the W-VO_2−x_@AA core-shell nanoparticles were characterized using X-ray diffraction (XRD, miniflex600, Cu Kα (λ = 1.5406 Å) radiation), and the degree of H-doping was evaluated based on the expansion of the (011) crystal plane. A Fourier transform infrared spectrometer (FT-IR, VERTEX 80v) was employed to analyze the chemical bonding in the nanoparticles; the presence of a characteristic absorption peak at 1398 cm^−1^, corresponding to the C-O-V bond, was used to determine whether chemical adsorption occurred between W-VO_2_ and AA. X-ray photoelectron spectroscopy (XPS, ESCALAB-Xi) was used to characterize the electronic density of the nanoparticles. The content of oxygen vacancies and the number of electrons injected by AA are assessed by analyzing the binding energies of the V 2p_3_/_2_ and V 2p_1_/_2_ peaks, thereby evaluating the effect of oxygen vacancies on the surface molecular coordination. The microstructure of the nanoparticles and composite films is examined using a field emission scanning electron microscope (FESEM, Regulus8100). A UV−VIS−NIR spectrophotometer (LAMBDA 750) with a temperature-controlling unit was used to obtain the optical property of the composite films.

## 3. Results and Discussions

### 3.1. Crystalline Structure and Morphology

**Figure 1:**
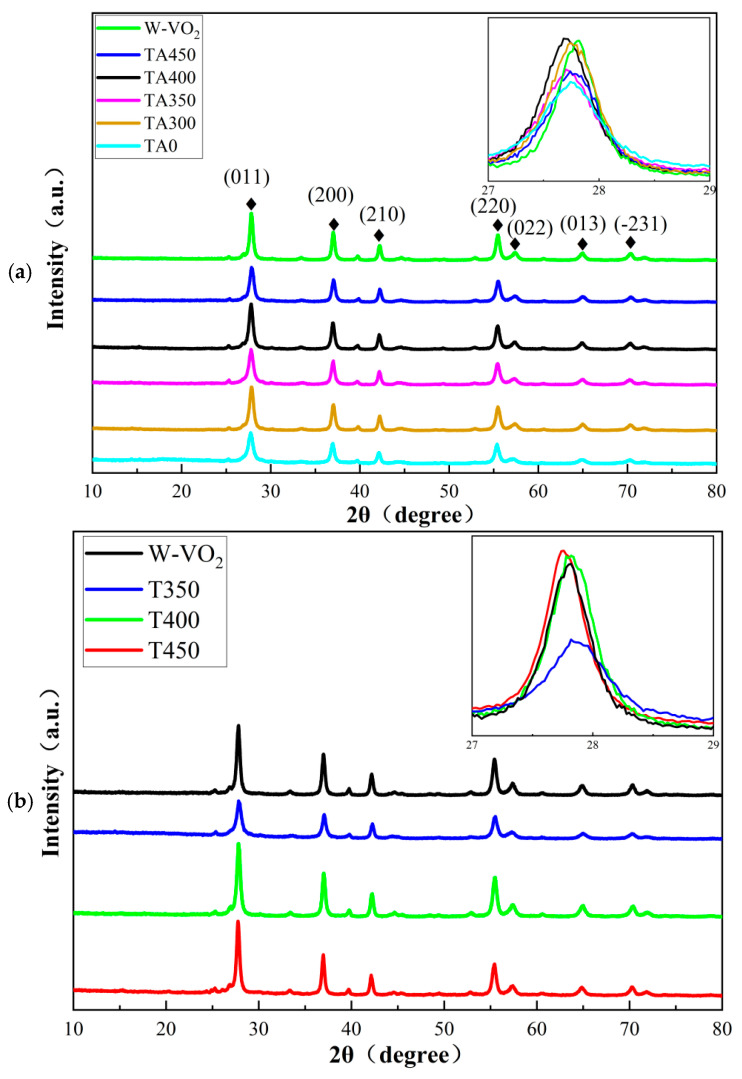
The crystalline of W-VO_2_ nanoparticle. (**a**) Connection with AA, (**b**) annealing without AA.

The surface molecular coordination between AA and W-VO_2−x_ nanoparticles prepared under different thermal annealing treatments always induces the variation in interplanar spacing of W-VO_2−x_. An XRD measurement was conducted on the following six samples: W-VO_2_, TA0, TA300, TA350, TA400, and TA450. The 2θ scanning range was from 10° to 80°, with a scanning rate of 5°/min. The XRD results are shown in Figure 1. It can be observed that all samples exhibit distinct diffraction peaks at 2θ = 27.82°, 39.74°, 42.24°, 55.5°, 57.48°, 64.92°, and 70.38°. By comparing these peaks to the standard XRD reference pattern (JCPDS#82-0661), it is evident that all the diffraction peaks correspond to VO_2_ (M), specifically to the (011), (200), (210), (220), (022), (013), and (-231) crystal planes [16,17], respectively. This indicates that the samples prepared through thermal annealing and hydrothermal treatment retain all the characteristic diffraction peaks, confirming that all samples are the VO_2_ (M). However, a closer comparison of the positions of the relevant diffraction peaks reveals slight shifts among different samples. Taking the (011) crystal plane as an example, the inset of Figure 1a presents the local XRD patterns within the range of 27–29° for all samples. From the inset, the diffraction peaks of TA0, TA300, TA350, TA400, and TA450 can be clearly observed. Compared to W-VO_2_, the diffraction peaks of these samples exhibit noticeable shifts toward lower angles, with reductions of 0.08°, 0.08°, 0.1°, 0.14°, and 0.08°, respectively. In particular, the peak of TA400 shifts from 27.82° to 27.68°, corresponding to a decrease of 0.14°. The shift of the diffraction peaks toward lower angles indicates an increase in the interplanar spacing along the (011) direction. The oxygen and incorporation of H^+^ can change the interplanar spacing along the (011) direction.

To investigate the effect of thermal annealing on the interplanar spacing, XRD are conducted on three samples (T350, T400, and T450) based on the previous tests. The results are shown in Figure 1b. The inset of Figure 1b presents the local XRD patterns in the 27–29° range for all samples. As shown in the inset, the peak position of W-VO_2_ is located at 27.82°, and the peaks of T350 and T400 are also at 27.82°, indicating that thermal annealing does not increase the interplanar spacing in the T350 and T400 samples. In contrast, the peak position of the T450 sample shifts to 27.76°, suggesting that thermal annealing results in increased interplanar spacing in the T450 sample. The T350 and T400 samples have undergone thermal annealing at relatively low temperatures and generated fewer oxygen vacancies, resulting in minimal changes in interplanar spacing. Since the diffraction peak of the T400 sample does not show a significant shift, it suggests that thermal annealing alone does not lead to an increase in interplanar spacing of the nanoparticles. Therefore, the change in interplanar spacing observed in the TA400 sample can be attributed to the surface molecular coordination effect. Specifically, the annealing temperature of 400 °C induced the formation of oxygen vacancies in the W-VO_2_ lattice, which enhanced the surface molecular coordination between AA and W-VO_2_. This intensified interaction led to greater electron injection into the W-VO_2_ lattice, thereby attracting more H^+^ into the lattice and ultimately increasing the interplanar spacing in the TA400 sample. In contrast, the diffraction peak of the TA450 sample shifts to a higher angle compared to TA400, indicating a lower level of H^+^ incorporation. This implies that a higher annealing temperature does not necessarily lead to increased H^+^ doping.

**Figure 2:**
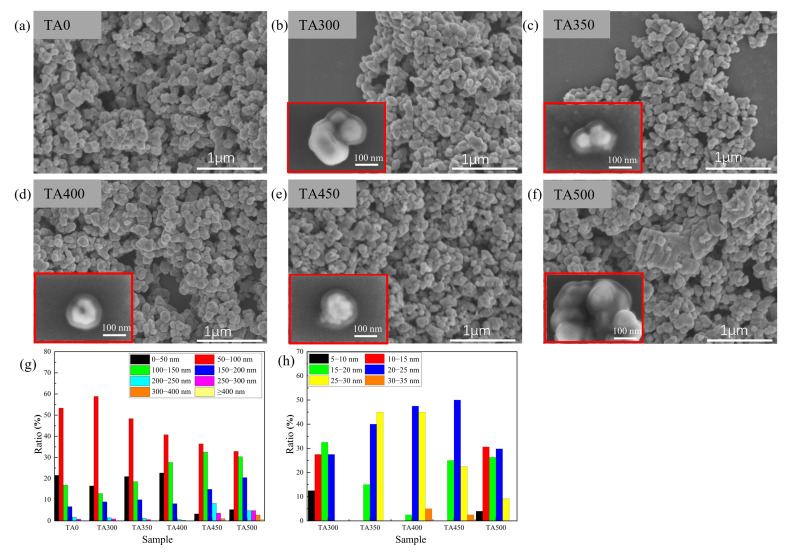
SEM image of surface morphology. (**a**–**f**) Annealing with different temperature, (**g**) statistical result of size of nanoparticles, (**h**) the statistical result of thickness of shell.

The morphology of the synthesized nanoparticles was analyzed and characterized using SEM, and the particle size was measured and statistically analyzed using Nano Measurer software (v1.2). The test and statistical results are shown in Figure 2. Due to the small size of the nanoparticles, no special dispersion treatment was applied, and no dispersant, such as PVP, was added, resulting in a certain degree of agglomeration among the nanoparticles. The microstructure of the W-VO_2_@AA nanoparticles without annealing treatment is shown in Figure 2a. The microstructures of W-VO_2−x_@AA nanoparticles synthesized by combining with AA after thermal annealing at different temperatures are shown in Figure 2b–f. The insets in Figure 2 display magnified images of the corresponding samples, highlighting the core-shell structure of W-VO_2−x_@AA. The average particle size and AA shell thickness of the samples were measured and statistically analyzed, as shown in Figure 2g,h. The nanoparticles are generally spherical in shape, and their particle size gradually increases with the rise in annealing temperature. Among them, the particle sizes of samples TA0, TA300, and TA350 are mainly distributed in the range of 0–100 nm. When the annealing temperature rises to 400 °C, the number of nanoparticles in the TA400 sample with particle sizes in the 50–100 nm range slightly decreases, while those in the 100–150 nm range slightly increase. When the temperature increases to 450 °C, larger block-like nanoparticles begin to appear in the sample, with a noticeable increase in the number of particles distributed in the 100–200 nm range. At 500 °C, the nanoparticle size increases further, and larger block-like particles can be clearly observed. Smaller spherical nanoparticles tend to adsorb onto the surfaces of larger block-like particles. Therefore, excessively high annealing temperatures lead to an increase in nanoparticle size and aggravate the agglomeration effect between nanoparticles, resulting in reduced dispersibility. Larger particle sizes and poorer dispersibility reduce the nanoparticles’ ability to absorb near-infrared light, ultimately leading to a decrease in the solar modulation rate of the films. The measured average AA shell thicknesses of the samples are shown in Figure 2h. The average AA shell thicknesses for the TA300, TA350, TA400, TA450, and TA500 samples are 18.3 nm, 23.7 nm, 27.6 nm, 23.5 nm, and 18.4 nm. Compared to the nanoparticles shown in the work of Liang et al. [14], the AA shell thicknesses of the TA350, TA400, and TA450 samples show a significant increase. This indicates that more AA molecules are adsorbed onto the surface of W-VO_2−x_, resulting in a thicker AA shell layer.

### 3.2. The Density of Electron of W-VO_2−x_@AA

**Figure 3:**
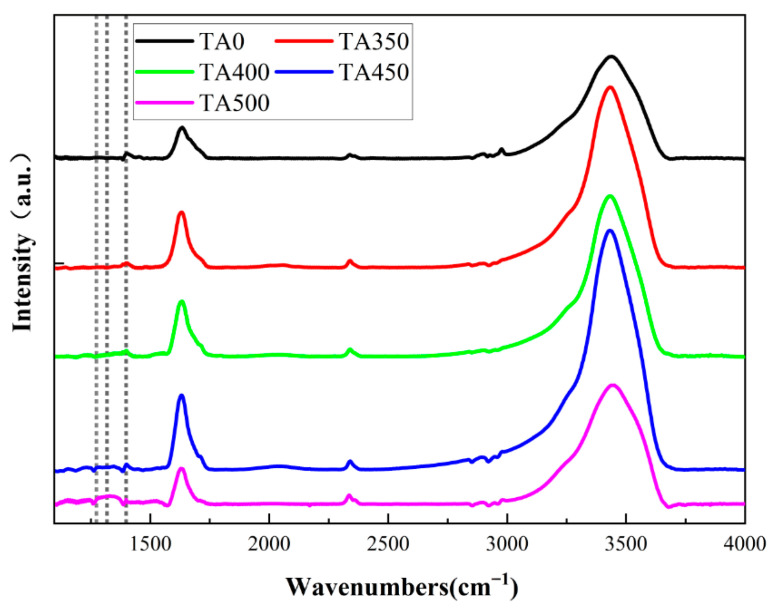
FTIR of W-VO_2−x_ nanoparticles connection with AA after annealing.

FTIR is used to characterize and verify the bonding interactions within the core-shell structure. The test results are shown in Figure 3. The black curve (TA0) represents the sample that is directly combined with AA without thermal annealing, while the other curves correspond to samples that are thermally annealed at 350 °C, 400 °C, 450 °C, and 500 °C, respectively, and then combined with AA via hydrothermal treatment. All samples exhibit a distinct absorption peak at 1398 cm^−1^, indicating the presence of C-O-V bonds in each case. Liang et al. [14] examined untreated W-VO_2_ and a sample prepared without the addition of acetic acid (S0). In the FTIR spectrum of W-VO_2_, no absorption peak is observed at 1398 cm^−1^, confirming that the original W-VO_2_ nanoparticles do not contain C-O-V bonds. In the S0 sample, an absorption peak corresponding to the C-O-V bond is observed at 1398 cm^−1^. Additionally, characteristic peaks at 1274 cm^−1^ and 1318 cm^−1^, attributed to the enolic C-O-H bending and stretching vibrations of AA molecules, are also detected. This indicates that a portion of the AA molecules underwent surface molecular coordination, forming C-O-V bonds, while the remaining AA molecules did not undergo chemical bond cleavage but were instead adsorbed onto the nanoparticles through intermolecular interactions. Among the samples tested, TA0, TA350, TA400, and TA450 all exhibit a distinct characteristic peak at 1398 cm^−1^, while no characteristic peaks are observed at 1274 cm^−1^ or 1318 cm^−1^. In contrast, the TA500 sample shows characteristic peaks at both 1274 cm^−1^ and 1318 cm^−1^, indicating the presence of a small amount of unreacted AA molecules. This is attributed to the suppression of the surface molecular coordination effect, leading to incomplete reaction. Therefore, when the thermal annealing temperature reaches 500 °C, the annealing process becomes unfavorable for the occurrence of surface molecular coordination.

**Figure 4:**
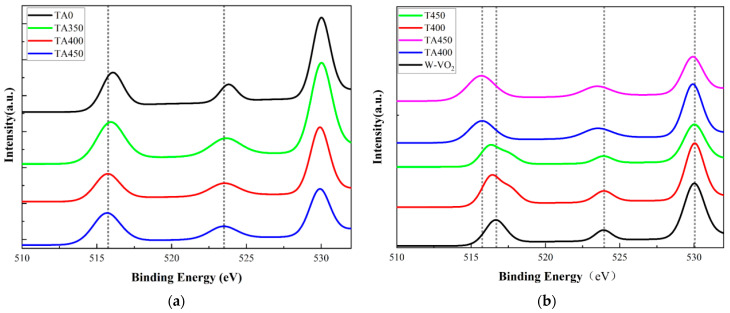
XPS spectra of (**a**) TA0, TA350, TA400, and TA450, (**b**) W-VO_2_, T400, T450, TA400, and TA450.

XPS was used to analyze the valence states of vanadium (V) in the samples, thereby characterizing the effects of thermal annealing and surface molecular coordination on the electron density of W-VO_2_ nanoparticles. An XPS measurement was conducted on the W-VO_2_, T400, T450, TA0, TA350, TA400, and TA450 samples, and the results are shown in Figure 4. The XPS spectra of the TA0, TA350, TA400, and TA450 samples are shown in Figure 4a, which shows V 2p core level peaks of W-VO_2_. The peak positions are normalized using the C 1s binding energy of 284.8 eV as a reference. Due to spin-orbit splitting, the characteristic peaks of V 2p_3/2_ and V 2p_1/2_ can be clearly observed [18], as shown in Figure 4a. For the TA450 sample, the V 2p_3/2_ peak appears at a binding energy of 515.65 eV, and the V 2p_1/2_ peak at 523.45 eV; for TA400, the V 2p_3/2_ and V 2p_1/2_ peaks are located at 515.7 eV and 523.5 eV, respectively; for TA350, the V 2p_3/2_ peak is at 515.9 eV and the V 2p_1/2_ peak at 523.6 eV; and for TA0, the V 2p_3/2_ and V 2p_1/2_ peaks are observed at 516.05 eV and 523.9 eV, respectively. According to the work by Silversmit G et al. [19], the binding energy of V 2p_3/2_ is 515.7 eV in V_2_O_3_, 516.2 eV in VO_2_, and 517 eV in V_2_O_5_. The V 2p_3/2_ peak of W-VO_2_ can be deconvolved into three main components according to the binding energy. The main composition or valence states of the sample can be deduced from the value of the binding energy. The V 2p_3/2_ binding energy of the TA0 sample is higher than 515.7 eV but lower than 516.2 eV, indicating the coexistence of *V*^3+^ and *V*^4+^ in the TA0 sample. Compared to TA0, the TA350, TA400, and TA450 samples exhibit lower V 2p_3/2_ binding energies, suggesting that the *V*^3+^ content in these three samples is higher than that in TA0.

The XPS results for W-VO_2_, T400, T450, TA400, and TA450 are shown in Figure 4b. The peaks in the spectrum corresponding to W-VO_2_ are located at binding energies of 516.65 eV and 523.95 eV. The V 2p_3/2_ binding energy of W-VO_2_ is 516.65 eV, which is higher than 516.2 eV (*V*^4+^) but lower than 517 eV (*V*^5+^). Therefore, the V in W-VO_2_ is primarily composed of *V*^4+^ and *V*^5+^. For the T400 spectrum, compare to W-VO_2_, a noticeable shift of the V 2p_3/2_ peak toward a lower binding energy is observed, with the binding energy decreasing to 516.45 eV. This indicates a reduction in the oxidation state of V during the thermal annealing process, accompanied by changes in electron density. The main chemical reaction occurring under vacuum conditions can be described by the following chemical equation [20]:

$$O_{o}^{x}↔V_{O}^{2+}+2e^{-}+\frac{1}{2}O_{2}$$

$$V^{4+}+e^{-}↔V^{3+}$$

Here, $O_{o}^{x}$ represents oxygen occupying an anion site, $V_{O}^{2+}$ denotes an oxygen vacancy, and *V*^4+^ and *V*^3+^ represent V ions in their original and reduced oxidation states, respectively. These chemical reactions indicate that the increase in *V*^3+^ content after thermal treatment is related to the formation of oxygen vacancies. Therefore, the decrease in the V 2p_3/2_ binding energy in the T400 sample is attributed to the reduction in the V oxidation state caused by the oxygen vacancies generated during thermal annealing. For the TA400 spectrum, both the V 2p_3/2_ and V 2p_1/2_ peaks exhibit a leftward shift, with a greater shift than that observed in the T400 sample. The binding energies of the V 2p_3/2_ and V 2p_1/2_ peaks decrease to 515.7 eV and 523.5 eV. Compared to the original W-VO_2_, the V 2p_3/2_ binding energy decreases by 0.95 eV, while the V 2p_3/2_ binding energy in the T400 sample decreases by 0.2 eV. The decrease in the V 2p_3/2_ binding energy of the T400 sample is attributed to thermal annealing. In the case of the TA400 sample, the reduction in V 2p_3/2_ binding energy results from both thermal annealing and electron injection. Specifically, thermal annealing accounts for a 0.2 eV decrease in the V 2p_3/2_ binding energy, while the electron injection induced by the surface molecular coordination effect of AA leads to an additional 0.75 eV decrease. Compared to W-VO_2_, the V 2p_3/2_ binding energy of the TA0 sample decreases by 0.6 eV. After thermal annealing, when W-VO_2_ reacts with AA, more electrons are injected from AA into the W-VO_2_ lattice through the C-O-V bonds, resulting in a greater proportion of *V*^4+^ being reduced to *V*^3+^.

For the W-VO_2_, T450, and TA450 samples, the V 2p_3/2_ binding energy of the T450 sample is 516.35 eV. Compared to T400, the lower V 2p_3/2_ binding energy of T450 indicates that the higher annealing temperature leads to the formation of more oxygen vacancies. The V 2p_3/2_ binding energy of the TA450 sample is 515.65 eV. Although the V 2p_3/2_ binding energy of TA450 is lower than that of TA400, it shows a 1 eV decrease compared to W-VO_2_. Among this, a 0.3 eV decrease is attributed to the oxygen vacancies, while the remaining 0.7 eV decrease results from electron injection via AA. This indicates that the additional oxygen vacancies do not further enhance the surface molecular coordination effect. An excessive number of oxygen vacancies may lead to the loss of oxygen atoms near the V atoms that bind with AA, preventing the formation of hydrogen bonds between AA and VO_2_, thereby reducing the overall stability of the composite structure.

### 3.3. The Thermochromic Performance

The resulted W-VO_2−x_@AA core-shell nanoparticles were dispersed into PVP and coated on the surface of glass (2.5 cm × 2.5 cm) to form smart window.

**Figure 5:**
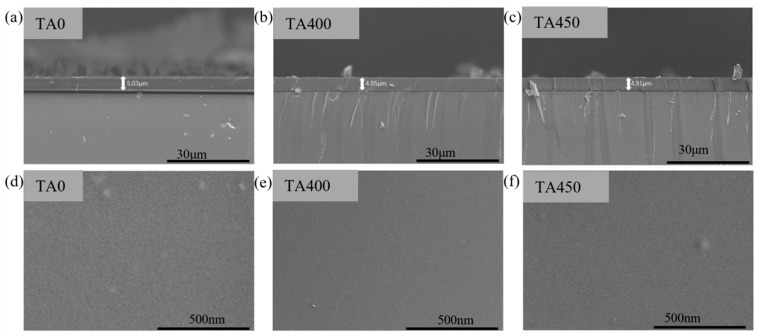
The surface morphology of W-VO_2−x_@AA/PVP, (**a**–**c**) cross-section of TA0, TA400, TA450, (**d**–**f**) surface microstructure of TA0, TA400, TA450.

The thickness and surface continuity of the composite films are also key factors affecting their optical properties. To eliminate the influence of film thickness, the cross-sectional and surface morphologies of the prepared films were characterized. The test results are shown in Figure 5, where Figure 5a–c present the cross-sectional images of the TA0, TA400, and TA450 samples after being spin-coated into films. According to the measurements, the thicknesses of samples TA0, TA400, and TA450 are 5.03 μm, 4.85 μm, and 4.91 μm. Since the sample thicknesses are maintained around 5 μm, the influence of film thickness on optical performance can be neglected. Figure 5d–f shows the surface images of the TA0, TA400, and TA450 samples after spin-coating. It can be seen from the images that the films are continuous and have smooth surfaces.

**Figure 6:**
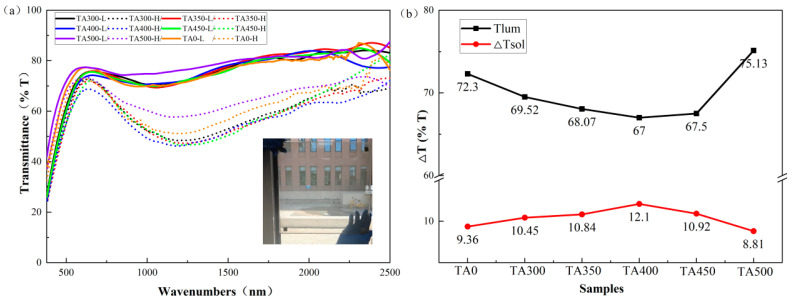
(**a**) The transmittance of samples at room temperature and high temperature, (**b**) the *T*_lum_ and ∆*T*_sol_.

The optical performance of the smart windows were characterized by UV-VIS-IR spectroscopy at 25 and 80 °C, and the results are shown in Figure 6.

It can be seen from Figure 6a that the transmittance has obviously decreased as the temperature increased from 25 to 80 °C. There is an obvious absorption peak in the transmittance curves of 80 °C, which is induced by the LSPR of metallic W-VO_2_. The LSPR absorption wavelength of sample TA0, TA300, TA350, TA400, TA450, and TA500 is 1180 nm, 1175 nm, 1175 nm, 1170 nm, 1175 nm, and 1180 nm.

The visual transmittance and solar modulation were calculated according to Figure 6a, as shown in Figure 6b. Sample TA0 is fabricated by unannealed W-VO_2_ and AA. When the annealing temperature is lower than 400 °C, the transmittance at 25 and 80 °C decrease with the annealing temperature, but the solar modulation increase. When the annealing temperature is higher than 400 °C, the case is reversed. The *T*_lum_ of sample TA0 is *T*_lum_ = 72.3%, and the *ΔT*_sol_ is 9.36%. The *T*_lum_ decreases to 67% after annealing at 400 °C, but the *ΔT*_sol_ increases to 12.1%. This change is related to the electron density induced by oxygen vacancy and AA [21]. The smart window based on sample TA450 has a similar *T*_lum_ but lower *ΔT*_sol_. This is related to the excessive oxygen vacancy induced by the higher annealing temperature, which suppresses the surface molecular coordination effect.

In order to evaluate the thermochromic performance, the *T*_lum_ and *ΔT*_sol_ of smart windows based on W-VO_2_ recently is summarized in Table 1.

### 3.4. The Effect of Oxygen Vacancy on Surface Coordination Effect

**Figure 7:**
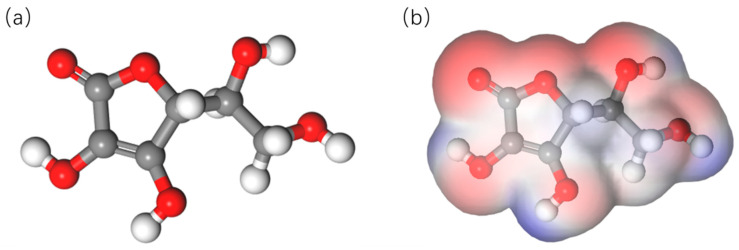
(**a**) Model of AA molecular structure, (**b**) electrostatic potential in AA.

In order to obtain the effects of oxygen vacancy on the surface molecular coordination effect, the electrons distribution on the AA molecules was calculated based on DFT. The model of the AA molecules was constructed, and the electrostatic potential of chemical bond was calculated; the results are shown in Figure 7a. The main chemical bonds are -C=C, -C=O, -OH, -C-O-C, and -CH_2_OH in AA molecules. When an AA molecule interacts with oxygen, the -C=C bond is oxidized to -C-C, the -C=O double chemical bond breaks and forms -C=O, and the H atom detaches in the -O-H bond. The electrostatic potential is shown in Figure 7b. It can be seen from the simulated results that the electron supply area is located near the bond of -OH, -C=O, and -C-O-C.

**Figure 8:**
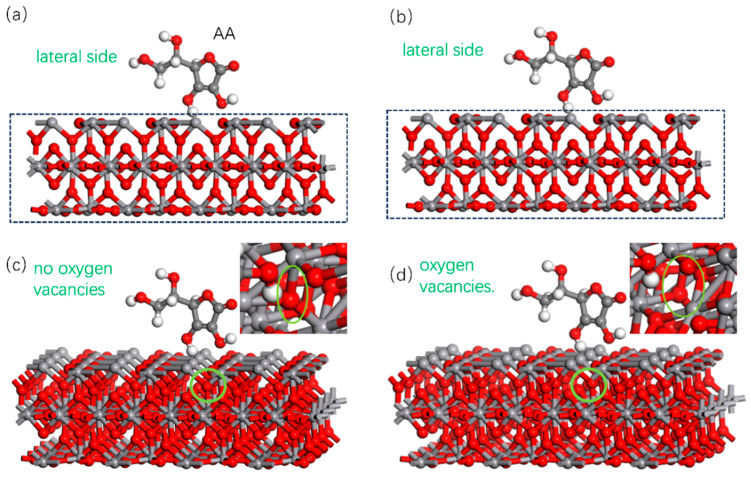
Absorption model (**a**) lateral side without oxygen vacancy, (**b**) lateral side with oxygen vacancy, (**c**) front view without oxygen vacancy, (**d**) front view with oxygen vacancy.

The absorbed model between AA molecules and W-VO_2_ and the simulation results are shown in Figure 8. In this model, the oxygen atom was deleted and linked with the V atom on the surface, and the V atom was considered as the absorb site. The position of oxygen vacancy is shown in the inset of Figure 8c,d. The environment temperature is 90 °C, so the structure of VO_2_ is a tetragonal rutile structure, because it is in the metallic state. The calculated absorb energy is −1.23 eV for W-VO_2_, but the calculated absorb energy is −1.93 eV for W-VO_2_ with the oxygen vacancy. It suggests that AA molecules are more easily absorbed on the surface of W-VO_2−x_, and the amount of AA is larger on W-VO_2−x_ than that on W-VO_2_. These simulated results are accordance with the experimental results.

**Figure 9:**
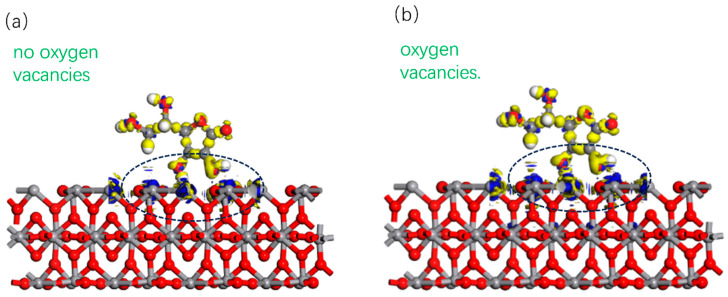
Charge density difference (**a**) without oxygen vacancies (**b**) with oxygen vacancies.

The results are shown in Figure 9. Figure 9a,b is without and with the oxygen vacancy, respectively. The yellow color denotes losing electrons, and blue color means accepting electrons. It can be seen from the figure that the area in blue is larger in W-VO_2_ with the oxygen vacancy than that without the oxygen vacancy. It suggests that the oxygen vacancy enhances the electron trapping ability of W-VO_2−x_ by introducing defects and reducing the oxidized state.

More AA molecules can be linked to W-VO_2−x_ through the oxygen vacancy. Additionally, more electrons flow from AA to W-VO_2−x_. The thermo-chromic performance of W-_2−x_/AA/PVP-based smart windows is affected by the AA molecules. The visual transmittance is affected by the refractive index and the electron concentration. The amount of AA molecules increases the thickness of the AA shell, which decreases the difference between W-VO_2_ and PVP. The decrease in the refractive index difference is helpful to increase the visual transmittance. The increase in the electron concentration increases the reflectance and decreases the visual transmittance. However, the effect of the electron concentration on visual transmittance is larger than that of the refractive index gradient. So, the combined action leads to the decrease in visual transmittance. The visual transmittance decreases.

For solar regulation, LSPR absorption is increased by the electron concentration, which increases the transmittance difference at room temperature and a high temperature. The increase in transmittance difference improves the solar regulation. However, as a comprehensive consideration, the electron concentration, by increasing the absorbed AA molecules through the oxygen vacancy, is conducive to improving the thermo-chromic performance.

## 4. Conclusions

Oxygen vacancies are regulated in W-VO_2_ nanoparticles by thermal annealing in a vacuum. Additionally, the AA molecules are adsorbed on the surface of W-VO_2−x_ nanoparticles through the hydrothermal method. The amount of absorbed AA molecules is increased by the oxygen vacancies on the surface of the W-VO_2−x_ nanoparticles. The electron concentration in W-VO_2−x_ nanoparticles is increased largely by electron flows from AA to W-VO_2−x_. The comprehensive thermo-chromic performance of smart windows based on the W-VO_2−x_/AA core-shell composite is enhanced by the improvement of solar regulation. The enhancement is caused by the increase in the transmittance difference at room temperature and the high temperature induced by the LSPR. This will enhance the application of W-VO_2_ nanoparticles in smart windows for energy-saving in building.

## Figures and Tables

**Table 1:** The Tlum and *ΔTsol* of the proposed W-VO2-x@AA/PVP composite films compared to reported studies.

Materials	*T_lum_*	*ΔT_sol_*	Refs
W-VO_2−x_@AA/PVP	67%	12.1%	this work
W-VO_2_@AA/PVP	70.52%	10.18%	[14]
SnO_2_/W-VO_2_/SnO_2_	53.45%	10.7%	[12]
SiO_2_/VO_2_ core-shell structures	27.8%	13.6%	[22]
ZrO_2_/V_1−x_W_x_O_2_	59%	6.0%	[4]
W-VO_2_	68.2%	9.8%	[23]
VO_2_ @ZnO core-shell nanoparticles	37.6%	17.2%	[24]

## Data Availability

Data will be made available on request.
